# Halotolerant Endophytic Bacteria Regulate Growth and Field Performance of Two Durum Wheat Genotypes with Contrasting Salinity Tolerance Potential

**DOI:** 10.3390/plants13091179

**Published:** 2024-04-23

**Authors:** Randa Albdaiwi, Rabea Al-Sayaydeh, Mohammad K. Al-Rifaee, Tareq Alhindi, Muhammad Ashraf, Ayed M. Al-Abdallat

**Affiliations:** 1 Department of Allied Medical Sciences, Zarqa University College, Al-Balqa Applied University, Al-Salt 19117, Jordan; 2 Department of Agriculture Sciences, Faculty of Shoubak College, Al-Balqa Applied University, Al-Salt 19117, Jordan; rabea.sayaydeh@bau.edu.jo; 3 National Agricultural Research Center (NARC), Amman 19381, Jordan; m.rifaee@narc.gov.jo; 4 Department of Biological Sciences, School of Science, The University of Jordan, Amman 11942, Jordan; t.alhindi@ju.edu.jo; 5 Hamdi Mango Center for Scientific Research (HMCSR), The University of Jordan, Amman 11942, Jordan; 6 Institute of Molecular Biology and Biotechnology, The University of Lahore, Lahore 54590, Pakistan; ashrafbot@yahoo.com; 7 Department of Horticulture and Crop Science, Faculty of Agriculture, The University of Jordan, Amman 11942, Jordan; a.alabdallat@ju.edu.jo

**Keywords:** biofertilizer, nutrient uptake, physio-biochemical processes, salinity stress, *Triticum turgidum* subsp. *durum*

## Abstract

Soil salinity hampers durum wheat plant growth and development at various stages. The detrimental effects of salinity on plant cellular and physiological processes necessitate strategies to alleviate its negative impact and improve overall crop yield. This study investigates the efficacy of plant growth-promoting rhizobacteria (PGPR) bacteria inoculation in mitigating salinity stress on two durum wheat genotypes with contrasting degrees of salinity tolerance (Tamaroi, salt-sensitive and Line 5004, salt-tolerant) under greenhouse and field conditions. For this purpose, two halotolerant-PGPR strains, *Pseudomonas jordanii* strain G34 and *Oceanobacillus jordanicus* strain GSFE11, were utilized for the inoculation. For the greenhouse experiment, the two selected genotypes were subjected to salinity at the flag leaf stage with continuous irrigation with a Hoagland solution supplemented with 50 mM NaCl. Field experiments were conducted across two locations with contrasting salinity levels over two growing seasons. At the end of both experiments, various parameters including total weight, spike weight, grain weight, spike number, spikelet number, grains per spike and thousand kernel weight were measured. The halotolerant PGPRs, *P. jordanii* strain G34 and *O. jordanicus* strain GSFE11, proved effective in alleviating salinity-induced adverse effects and enhancing growth under greenhouse and field conditions. However, bacterial inoculation significantly improved growth in the salt-sensitive genotype and such effects were not observed in the tolerant genotype, emphasizing genotype-specific responses. Notably, inoculation with *O. jordanicus* increased Na^+^ and Ca^2+^ uptake in the salt-tolerant “Line 5004” without hindering growth, suggesting one of its potential mechanisms for salt tolerance. This research demonstrates the potential of halotolerant-PGPR inoculation in enhancing durum wheat production in saline environments, but also underscores the importance of understanding genotype-specific responses for tailored interventions.

## 1. Introduction

Soil salinity poses a major threat to crop productivity worldwide, particularly in arid and semi-arid regions of the world [1]. High rates of evapotranspiration, improper drainage and limited leaching of mineral salts from the soil surface result in increased salinity levels in arid and semiarid regions [2]. Under saline regimes, the yield of any given crop is significantly reduced [3]. In this perspective, soil salinity adversely affects almost all stages of plant development [4]. Furthermore, the inhibitory effect of salinity affects many plant physio-biochemical and molecular processes leading to impaired plant growth and subsequent reduction in crop production [5]. Thus, there is a need to assuage the deleterious effects of salt stress across these crucial phases, consequently resulting in enhancement of the overall crop yield. 

Many cereal crops are categorized as salt-sensitive [6]. This sensitivity highlights the vulnerability of cereals to adverse effects resulting from salinity and the need to address salt stress management in cereal cultivation. Soil salinity is known to cause detrimental effects on morphological and physiological, and biochemical processes [7]. Salinity can affect the wheat plant development by reducing water and nutrient uptake, photosynthetic capacity, enzymatic activities, and subsequently overall crop yield [8]. Although salinity affects all stages of wheat growth, the most sensitive development stages include the early growth stages [9]. It has been widely reported that salt stress effects vary from species to species or cultivar to cultivar [10,11,12]. In durum wheat (*Triticum turgidum* subsp. *durum*), high salt levels have a more prominent influence on growth compared to its other relative species as well as cereals [13,14]. Bread wheat (*T. aestivum*) is ranked as moderately salt-tolerant, whereas durum wheat as salt-sensitive. The salt sensitivity of the latter wheat type is ascribed to its inefficient exclusion of Na^+^ from its tissues [15]. The ability to exclude Na^+^ in the durum wheat was improved by the introduction of high-affinity K^+^ transporters, *TmHKT1;4-A2* (*Nax1*) and *TmHKT1;5-A* (*Nax2*) from *Triticum monococcum*, that significantly improved salinity tolerance under field conditions [16,17]. The introduction of *Nax1* and *Nax2* genes through breeding programs allowed the development of durum wheat lines with improved salinity tolerance [18,19].

Besides plant breeding, several approaches have been used to manage the deleterious effects of soil salinity on wheat [20]. For instance, soil amendments such as biochar application improved wheat productivity under saline conditions [21]. Irrigation management using drip irrigation was effective in managing salinity and improving wheat performance [22]. The salt tolerance trait in different plants can be also increased by using eco-friendly approaches, including the exploitation of halotolerant microorganisms that interact with plant roots [23]. For example, plant growth-promoting rhizobacteria (PGPR), have useful influences on plants, increasing the overall yield of crops and helping to mitigate the effects of abiotic stresses through a variety of mechanisms [24]. Plant growth-promoting rhizobacteria can enhance growth by changing the levels of key hormones, e.g., ethylene production decreases within the plant body through the activity of the 1-aminocyclopropane-1-carboxylate (ACC) deaminase enzyme or IAA production [25]. Under saline conditions, endophyte strains expressing ACC deaminase activities were found to ameliorate salt stress conditions and improve growth by reducing the levels of ethylene in stressed plants [26]. Inoculating wheat with PGPR that produces ACC deaminase improved growth under saline regimes by decreasing the generation of ethylene, a stress-related hormone in plants [27]. 

This study evaluated the potential of two halotolerant endophytic bacterial strains, *Pseudomonas jordanii* strain G34 (NCBI accession: PRJNA991609; Albdaiwi, unpublished data) and *Oceanobacillus jordanicus* strain GSFE11 (NCBI accession: PRJNA753452 [28]), in mitigating the salinity-induced adverse effects on durum wheat growth. Halotolerant-PGPR strains have demonstrated the potential to reduce salinity stress in durum wheat seedlings [28,29]. The experimental design of our research involved conducting both greenhouse and field trials to assess the effects of bacterial inoculation on the growth and yield of two durum wheat genotypes under varying conditions. The deployed experimental approach facilitated a thorough examination of bacterial effects on durum wheat across diverse environmental conditions. For this purpose, the effects of inoculating two durum wheat genotypes with contrasting salinity tolerance (Tamaroi, salt-sensitive and Line 5004 carrying the *Nax2* gene, salt-tolerant) with these two PGPR strains was studied. Agronomic and growth-related traits, such as total weight (TW), GY (grain yield) thousand kernel weight (TKW) were considered. However, certain parameters, such as grain quality properties and nutrient content, were assessed separately in each experiment. This study contributes valuable insights regarding the potential of PGPR inoculation as a strategy to enhance durum wheat productivity under saline conditions.

## 2. Results

### 2.1. Greenhouse Experiment 

The analysis of variance (ANOVA) of the greenhouse experiment showed a significant effect (*p* ≤ 0.05) among salinity treatments for all agronomic traits, with the exception of spike number (SN) and kernel length (KL) (Table S1). Furthermore, significant variations among salinity treatments were observed on nutrient levels and the potassium to sodium ratio (K^+^:Na^+^). For the genotypic effect, significant variations were detected in five agronomic traits, specifically in grain weight (GW), grain per spike (GpS), thousand-kernel weight (TKW), kernel area (KA) and kernel width (KW), while for nutrient levels, significant variations for all measurements were observed expect for Mg^2+^ content (Table S1). The main effect of bacteria was significant for total plant weight (TPW), GW, spike weight (SW), spikelet number (SplN), TKW, KA, KW, KL, Na^+^ and Ca^2+^. For the interactive effect bacteria × genotype, significant differences were detected for GW, SW, KA, KW, Na^+^, Mg^2+^ and Ca^2+^ (Table S1). For the interactive effect between treatments, genotypes and bacteria, significant differences were detected only in TKW, KA, KW and Na^+^ level (Table S1). For the coefficient of variations (CV), the values varied for the tested traits where KW showed the least variation percentage (2.00%), while SN showed the highest variability with a CV of 13.10% (Table S1).

The negative effects of salinity treatment were evident in all agronomic traits, with a marked decline in mean values under saline regimes when compared to those under non-saline treatment (Table S2). For instance, the mean value of GW was significantly lower (11.54 g) compared to those under the non-saline treatment (15.90 g). For nutrients’ levels, salinity substantially increased the level of Na^+^ when compared to that in the non-saline conditions, while a significant reduction was observed for K^+^, Ca^2+^ and Mg^2+^ under saline conditions (Table S2). For genotypic effect, marked differences were recorded for GW, GpS, TKW, KA, and KW, where Line 5004 showed higher mean values for GW and GpS and lower mean values for the remaining traits (Table S2). For nutrient levels, marked differences were noted for K^+^, Na^+^, K^+^:Na^+^ and Ca^2+^, where Tamaroi had a higher mean value of Na^+^ and Line 5004 showed high mean values for the remaining nutrients (Table S2). For the bacteria effect, lower mean values were observed in non-inoculated plants for several agronomic parameters including TPW, GW, SW, SplN, TKW, KA, KW and KL. For nutrient levels, inoculated plants with *O. jordanicus* had significantly higher mean values for Na^+^ and Ca^2+^ when compared with other types of bacteria and the control (Table S2). 

**Figure 1:**
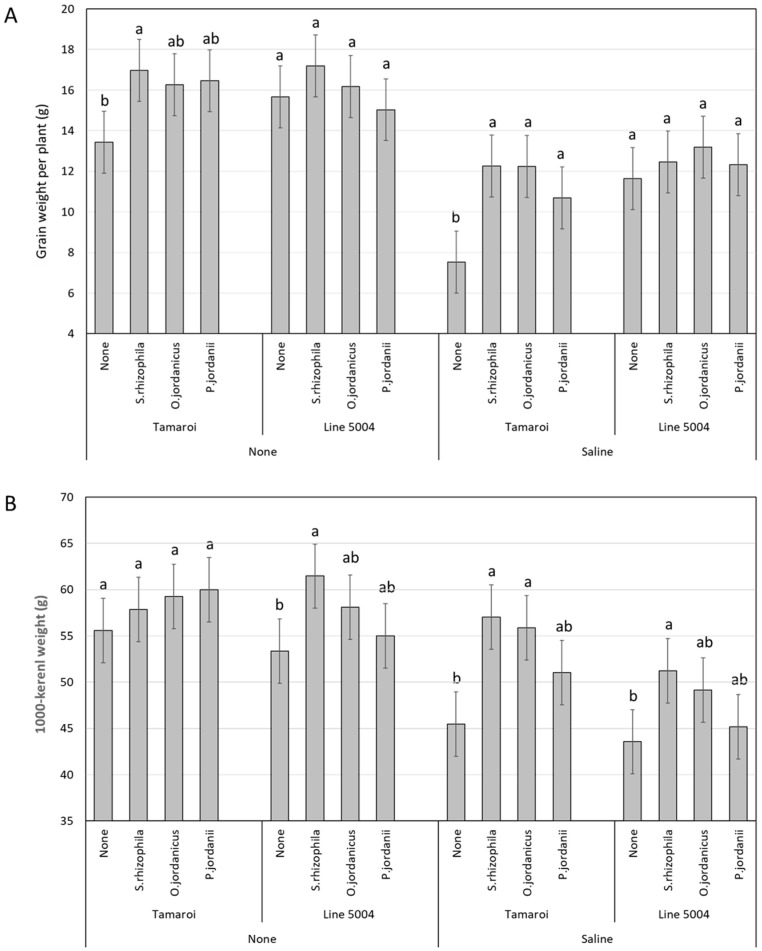
(**A**) Mean values of grain weight for two durum wheat genotypes (Tamaroi and Line 5004 (carries *Nax2*)) grown under two different salinity conditions and inoculated with different types of PGPR. (**B**) Mean values of thousand-kernel weight for two durum wheat genotypes (Tamaroi and Line 5004 (carries *Nax2*)) grown under two different salinity conditions and inoculated with different types of PGPR. Bars represent LSD values at *p* ≤ 0.05 to compare means for the combined analysis, while letters were used to compare each genotype within the bacteria × salinity treatment combination based on LSD value at *p* ≤ 0.05.

For the interactive effect bacteria × genotype, comparison of the mean values of each tested genotype inoculated with different types of bacteria at the same salinity treatments showed a marked suppression in GW in non-inoculated Tamaroi under saline conditions compared with Tamaroi inoculated with different types of bacteria (Figure 1A). Interestingly, Line 5004, carrying the *Nax2* gene, did not show any significant differences between different types of bacteria irrespective of salinity treatments. For the interactive effect bacteria × genotype × salinity, a significant reduction in mean TKW values were observed in non-inoculated Tamaroi and Line 5004 plants under saline conditions when compared with *Stenotrophomonas rhizophila*-inoculated plants (Figure 1B). Under salinity, *O. jordanicus*-inoculated Tamaroi plants displayed higher TKW values compared to those in non-inoculated Tamaroi plants, which were not significantly different from the *P. jordanii*-inoculated plants. 

**Figure 2:**
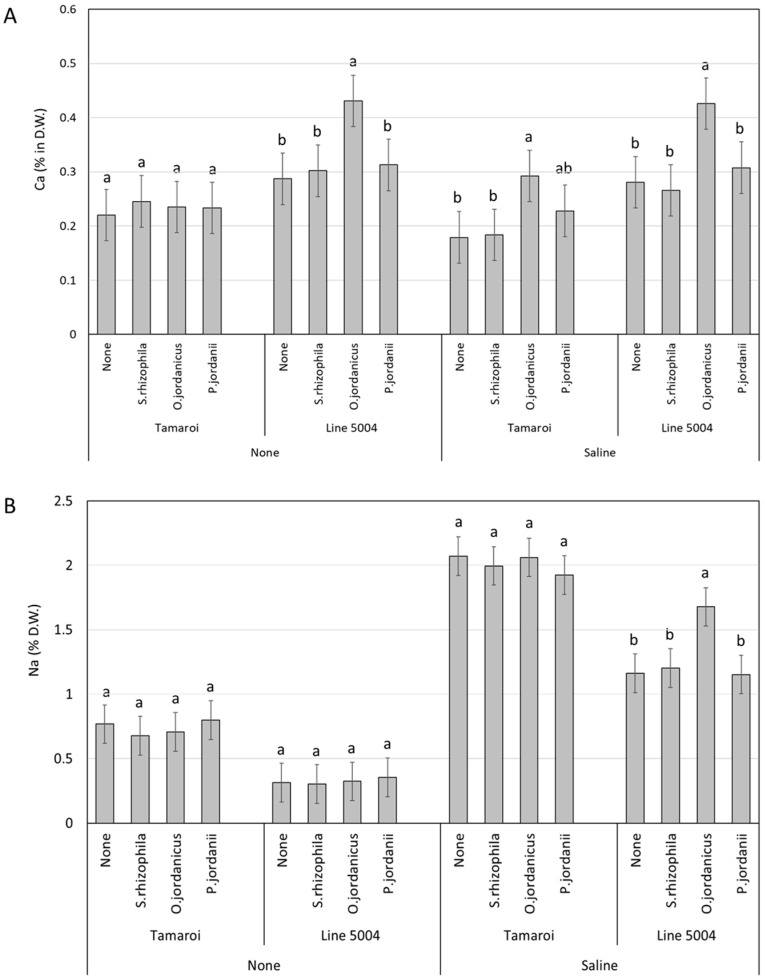
(**A**) Mean values of Ca^2+^ levels (%) for two durum wheat genotypes (Tamaroi and Line 5004 (carries *Nax2*)) grown under two different salinity conditions and inoculated with different types of PGPR. (**B**) Mean Na^+^ concentration (%) for two durum wheat genotypes (Tamaroi and Line 5004 (carries *Nax2*)) grown under two different salinity conditions and inoculated with different types of PGPR. Bars represent LSD values at *p *≤ 0.05 to compare means for the combined analysis, while letters were used to compare each genotype within bacteria × salinity treatment combination based on LSD at *p *≤ 0.05.

For nutrient levels, comparison of the mean values of Ca^2+^ in each tested genotype inoculated with different types of bacteria at the same salinity treatment showed a significant increase in the Ca^2+^ level in *O. jordanicus*-inoculated Line 5004 plants irrespective of salinity treatment compared to other bacteria treatments (Figure 2A). In contrast, Tamaroi plants inoculated with *O. jordanicus* under salinity treatment showed prominent differences with non-inoculated and *S. rhizophila*-inoculated plants; however, no clear difference was observed when compared to *P. jordanii* inoculated plants. For Na^+^, the interactive effect of bacteria × genotype × salinity displayed a marked enhancement in the Na^+^ levels in the inoculated *O. jordanicus* Line 5004 plants under saline regime compared to other bacteria treatment (Figure 2B). 

**Figure 3:**
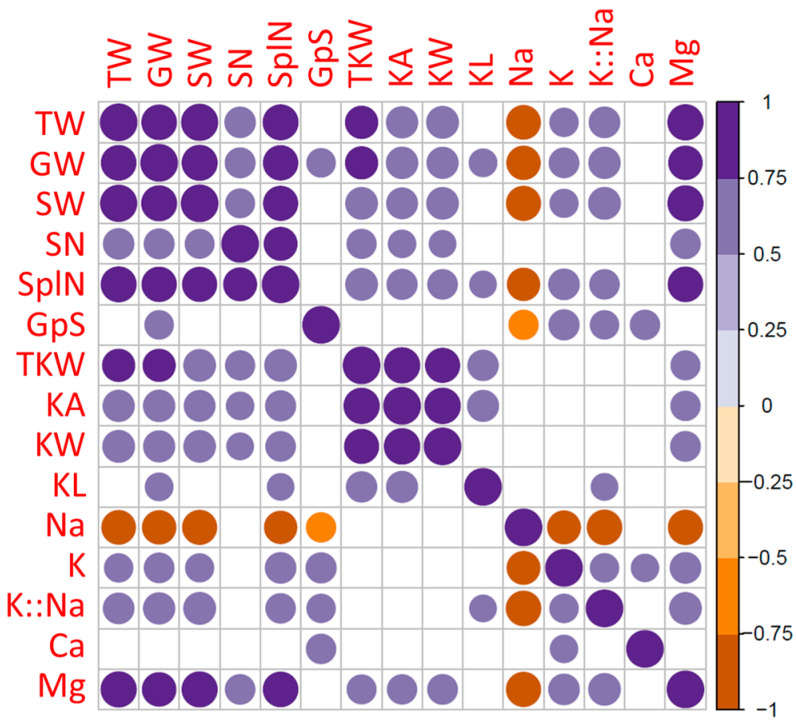
Pearson’s coefficients and pairwise correlations among traits in two durum wheat genotypes (Tamaroi and Line 5004 (carries *Nax2*)) grown under two different salinity conditions and inoculated with different types of PGPR. (Blank squares denote non-significance at *p* < 0.05 level).

For pairwise correlation analysis, strong significant positive correlations (*p* < 0.05) were detected among several agronomic traits such as GW with TPW, SW, SN, GpS and SplN and TKW (Figure 3). Furthermore, K^+^ and Mg^2+^ levels showed significant positive correlations with TPW, GW, and SW, while Mg^2+^ alone showed significant positive correlations with TKW, KA and KW traits. Negative correlations were only observed between the Na^+^ level and TW, GW, SW, GpS, SplN, K^+^ levels, K^+^:Na^+^ and Mg^2+^ levels (Figure 3). No correlation was detected between Na^+^ level and TKW and kernel traits.

### 2.2. Field Experiment

The weather data for Jordan University (JU) and Al-Khalediyah (KD) showed that JU experienced higher rainfall intensity than the average in both seasons, while KD had rainfall lower than the average in 2021 with 102.0 mm [30]. In 2021, both locations showed less precipitation in April and May imposing drought conditions in JU, while in KD, supplementary irrigation from a saline water source was provided to the field trials for both growing seasons. For temperatures, January was the coldest month at both sites irrespective of the season and the highest average maximum temperature was recorded in KD in May 2021. The 2021 season was notably warmer than usual, contrasting with the wetter 2020 season for both JU and KD [30].

The combined analysis of variance (ANOVA) of the field trial displayed a significant effect (*p* ≤ 0.05) among the environments (each location and year combination was considered a single environment) for all agronomic and quality traits (Table S3). Furthermore, significant variation among the genotypes were observed for straw weight (StW), harvest index (HI), TKW, KW and KL and for all quality traits except for dry matter (DM). For the bacteria effect, significant variations were detected in eight agronomic traits, specifically in total weight (TW), GW, HI, SN, GpS, TKW, KA and KW, while for quality parameter, significant variations were observed only for protein content and DM (Table S3). For the interactive effect between bacteria and genotypes, significant variations were observed for TW, GW, StW, SN, TKW, KA, KW, KL and protein content. For the combined environment, genotypes and bacteria interaction, significant differences were detected only in TKW and KA (Table S3). For the coefficient of variations (CV), the values varied for the tested traits where DM showed the least variation percentage (0.30%), while GpS showed the highest variability with a CV of 11.60% (Table S3).

The mean values of the tested genotypes for all traits recorded in each environment are presented in Table S4. At the environment level, the highest mean value for GY was recorded in JU20 and it was significantly different from other tested environments (JU21, KD20 and KD21) (Table S4). The same trend was observed for TW, StW and SN with JU20 producing the highest mean value when compared to other environments (Table S4). Interestingly, JU20 produced significantly the lowest mean value for protein content in harvested grains when compared to other tested environments. At the genotype level, significant differences were observed for TKW and kernel-related traits (except KW) with Tamaroi producing a significantly higher mean value for TKW compared to Line 5004 (Table S4). In addition, clear variations were observed for quality-related traits with Line 5004 producing significantly higher mean values for protein, fat, fiber and ash contents when compared to Tamaroi (Table S4).

**Figure 4:**
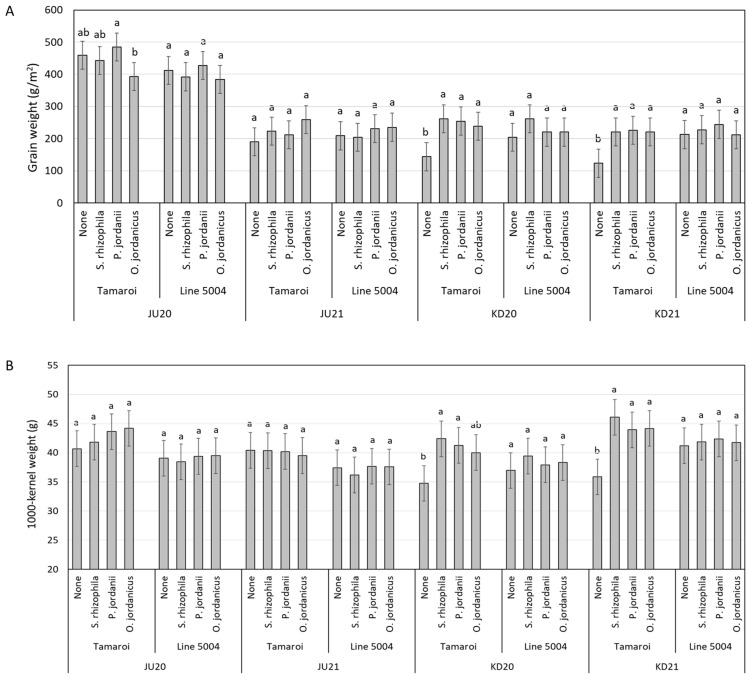
(**A**) Mean values of grain weight for two durum wheat genotypes (Tamaroi and Line 5004) grown across four environments (JU20, JU21, KD20 and KD21) and inoculated with different types of PGPR. (**B**) Mean values of 1000-kernel weight for two durum wheat genotypes (Tamaroi and Line 5004 (carries *Nax2*)) grown across four environments (JU20, JU21, KD20 and KD21) and inoculated with different types of PGPR. Bars represent LSD values at *p *≤ 0.05 to compare means for the combined analysis, while letters were used to compare each genotype within bacteria × environment combination based on LSD value at *p *≤ 0.05.

For the interactive effect of bacteria × genotypes, significant reductions in GW mean values in non-inoculated Tamaroi were observed in saline environments (KD20 and KD21) when compared to the inoculated plants (Figure 4A and Figure S1). Similar to the greenhouse experiment, no significant differences were observed for Line 5004 GW among the bacteria treatments irrespective of salinity treatments. For the interactive effect of bacteria × genotypes × environment, a marked decline in TKW values were observed in non-inoculated Tamaroi under saline field conditions (Figure 4B). 

**Figure 5:**
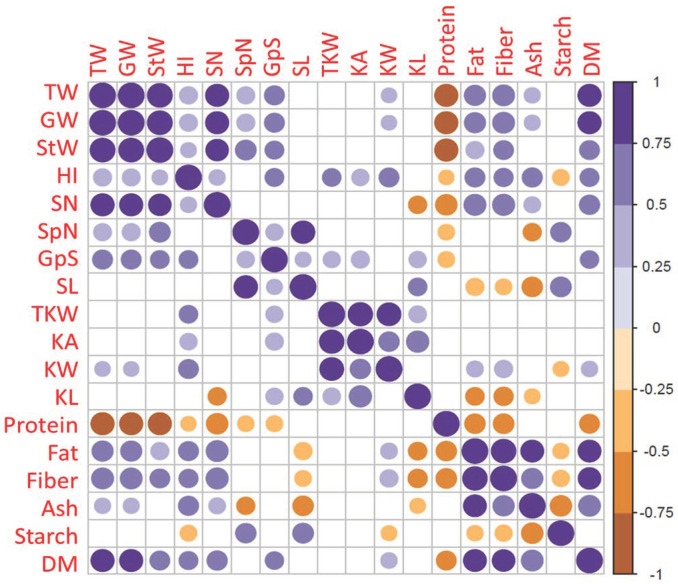
Pearson’s coefficients and pairwise correlations among traits in two durum wheat genotypes (Tamaroi and Line 5004 (carries *Nax2*)) grown across four environments (JU20, JU21, KD20 and KD21) and inoculated with different types of PGPR. (Blank squares denote non-significance at *p *< 0.05 level).

For pairwise correlation analysis, strong significant positive correlations (*p *< 0.05) were detected between GW and other yield components-related traits such as TW, SW, StW, HI, SN, GpS and SplN (Figure 5). Furthermore, GW showed significant positive correlations with fat, fiber, ash and DM levels. For TKW, significant positive correlations were observed with HI and GpS and with other kernel-related traits (KA, KW and KL). Negative correlations were observed between the protein level and TW, GW, StW, HI, SN, GpS and SplN (Figure 3).

## 3. Discussion

In this research, greenhouse and field trials were performed to demonstrate the effect of PGPR bacteria inoculation on alleviating the detrimental impact of salinity on two durum wheat genotypes with contrasting salinity tolerance response. Salinity significantly reduced various agronomic traits, including TPW, GW, and TKW that were associated with higher Na^+^ uptake. These findings agree with previous studies reflecting the negative effects of high salt and stress on durum wheat [3,31]. Regarding genotypic effect, Tamaroi was significantly affected by salinity under controlled conditions and showed higher Na^+^ accumulation in its leaf tissues compared to Line 5004 (carrying the *Nax2*) and this is in total agreement with the findings recorded earlier by James et al. 2012 [17]. Furthermore, Line 5004 was found to exhibit higher GW and GpS compared to Tamaroi, but generally lower values for other traits and this might suggest that the *Nax2* gene may confer tolerance to salinity for specific components of the yield.

In this study, the use of two halotolerant PGPRs (*P. jordanii* strain G34 and *O. jordanicus* strain GSFE11) proved useful in mitigating the negative effects of salinity, thereby stimulating the growth of the tested durum wheat genotypes, which is in general agreement with previous findings [29,32]. Inoculation with the halotolerant-PGPR strains significantly improved several agronomic traits (TPW, GW, etc.) compared to non-inoculated controls. *Pseudomonas* spp. were reported to ameliorate high salt stress in wheat by significantly increasing grain yield compared to that in non-inoculated under controlled conditions [33]. In another study, *Oceanobacillus* sp. strain 76 was found to enhance wheat growth under saline conditions with clear positive effects on seed germination and seedling growth [34]. To address salinity stress in wheat, future studies should consider exploring the effectiveness of halotolerant-PGPR inoculation as biofertilizers for growing both sensitive and tolerant wheat genotypes on saline environments. Such research can inform the development of tailored, PGPR-based strategies for sustainable wheat production in saline environments [32].

In both experiments, bacteria inoculation was successful in improving the GY, quality and the growth of the sensitive durum wheat genotype, while no marked effect was observed in the tolerant genotype. This highlights the genotype-specific responses to PGPRs in mitigating salinity effects. Line 5004 (with *Nax2*) showed less variation in GW under different bacterial inoculations and salinity treatments, suggesting potential mechanisms independent of bacterial intervention. Conversely, Tamaroi displayed significant improvements in GW and TKW with PGPR inoculation, particularly under salinity stress. Similar to our findings, PGPR inoculation of two barley cultivars with contrasting salinity tolerance response significantly improved growth and yield parameters in the sensitive genotype, but it had no marked influence on the salinity-tolerant genotype [35]. However, in another study, a salt-resistant wheat variety inoculated with different types of bacteria showed better growth under salinity conditions compared to a salt-sensitive genotype [36]. It is worth mentioning that the observed genotype-specific responses and contrasting findings from other studies underscore the complexities of plant-microbe interactions under salinity conditions. 

Interestingly, Line 5004 (with *Nax2*) inoculated with *O. jordanicus* showed higher levels of Na^+^ compared to those in the non-inoculated and other bacteria types without affecting its growth. The obtained results suggest that *O. jordanicus* may enhance Na^+^ and Ca^2+^ uptake, potentially influencing plant growth and stress tolerance. Similar findings were recorded elsewhere [37], where higher shoot Na concentration was observed in the inoculated *Salicornia* sp. plants with *Staphylococcus* sp. E14 compared to that in non-inoculated plants. In addition, *O. jordanicus* was capable of accumulating a higher level of Ca^2+^ in the leaves of Line 5004 (*Nax2*) compared to the other bacterial treatment. Such an effect was observed previously in chickpea plants inoculated with *Bacillus subtilis* (BERA 71) under high salt stress conditions [38]. Such increase in Ca^2+^ uptake coupled with higher Na^+^ uptake in Line 5004 (*Nax2*) inoculated with *O. jordanicus* might explain the absence of the negative impact of salinity on the inoculated plants. It is well documented that the translocation of Ca^2+^ is more affected by Na^+^, particularly in wheat-sensitive genotypes resulting in greater salt sensitivity and growth inhibition [39]. On the other hand, a rapid increase in Ca^2+^ levels in the cytosol was observed after exposure to salt stress and such an increase was associated with tolerance mechanisms that are coordinated by Ca^2+^ signaling [40]. Under salt stress, the application of Ca^2+^ significantly enhanced wheat plant growth, which was associated with improved photosynthetic activity, increased proline concentration and the activities of antioxidant enzymes [41]. The elevated Na^+^ and Ca^2+^ uptake in *O. jordanicus*-inoculated Line 5004 (*Nax2*) plants, without hindering growth, suggests possible mechanisms involving regulation of ions or molecular stress–response pathways. Further investigation into the specific mechanisms by which *O. jordanicus* alleviates stress in plants containing high levels of Na^+^ could be explored.

The study sheds light on the potential of halotolerant-PGPR inoculation as a means to assuage salinity stress in durum wheat. The findings of this study are particularly encouraging with respect to the improved salinity tolerance in the salt-sensitive genotypes, where significant improvements in several agronomic traits were observed with bacterial inoculation, especially under saline conditions. These findings, along with the observed higher Na^+^ and Ca^2+^ levels in the *O. jordanicus*-inoculated plants suggest unknown prospective mechanisms to alleviate the negative effect of salinity in durum wheat. However, the lack of a significant response in the salinity-tolerant Line 5004 suggests that PGPR effectiveness might be genotype-specific, necessitating further investigation into genotype–bacteria interactions in response to salinity. These findings highlight the need for in-depth molecular studies to elucidate the exact mechanisms by which *O. jordanicus* confers salinity tolerance in different wheat genotypes. 

Overall, this investigation offers significant insights into the potential of the two described PGPR strains as biofertilizers for mitigating salinity stress in durum wheat. Further research on elucidation of underlying mechanisms and genotype-specific interactions can be beneficial for developing practical applications to enhance wheat production in saline environments.

## 4. Materials and Methods

### 4.1. Bacteria Strains

Two halotolerant endophytic strains, *Pseudomonas jordanii* strain G34 (NCBI accession: PRJNA991609; Albdaiwi, unpublished data) and *Oceanobacillus jordanicus* strain GSFE11 (NCBI accession: PRJNA753452) [28], have been identified in the root tissue of field-grown durum wheat plants [29] (Albdaiwi, unpublished data). These strains exhibited growth-promoting activity and were able to produce indole-3-acetic acid (IAA) and enhanced drought and salinity tolerance in durum wheat. Notably, *O. jordanicus* strain GSFE11 demonstrated nitrogen-fixing capabilities and siderophores production. A reference endophytic strain, *Stenotrophomonas rhizophila* (ATCC^®^ DSM 14405T), known for its growth-promoting activity, was used as a positive control [42]. All strains were cultured on the nutrient agar (NA) medium (Thermo Fisher Scientific Oxoid Ltd., Basingstoke, UK) containing 1% NaCl at 28 °C for 72 h. Thereafter, the cultures were used to prepare the bacteria inoculum as described previously [29].

### 4.2. Plant Material and Bacterial Inoculation

In this study, two durum wheat genotypes with contrasting salinity tolerance were used. The first genotype, Tamaroi, a salt-sensitive cultivar, was originally from Australia. The second genotype, Line 5004, a salt-tolerant BC4F2 homozygous line possessing the *NAX2* (*TmHKT1;5-A*) gene that was taken from the backcrossing line 149 (*TmHKT1;5-A*) with Tamaroi as a recurrent parent [43].

For bacterial inoculation, seeds from both lines were surface sterilized with 70% ethanol solution for one min, subsequently the seeds were washed three times with sterile distilled water and then they were soaked in sodium hypochlorite solution (1.5%). Finally, these treated seeds were washed six times with sterile water. Afterwards, the sterilized seeds were placed in bacterial suspension as illustrated previously [29].

### 4.3. Greenhouse Experiment

The greenhouse experiment took place at the School of Agriculture, The University of Jordan, starting on 1 November 2019, and continuing until the plants attained the harvest adult stage (Zadoks scale: GS87). Two seeds from each genotype, either inoculated with bacteria or left without inoculation (negative control), were sown in plastic pots, each with a size of 10 L. These pots were filled with eight kg of acid-washed sand, watered to reach saturation, and then placed in greenhouse conditions to initiate seed germination. Initially, each pot was watered until it reached saturation by adding 1000 mL of distilled water. The pots were then left to reach pot capacity, with an estimated weight of 8.5 kg. Pot capacity was subsequently maintained by daily irrigation with 250 mL of water until the full emergence of the seedlings and until they reached the one-leaf stage. A thinning process was then conducted to retain one seedling per pot and ensure homogeneity among the remaining treatments. The seedlings were irrigated twice a week with fixed volumes (250 mL) of 1× Hoagland’s nutrient solution (Sigma-Aldrich, Darmstadt, Germany) to maintain moisture content at pot capacity until the initiation of salinity stress treatment, which began at the emergence of the flag leaf (Zadoks scale: GS37). At the flag leaf stage, two 1× Hoagland’s nutrient solutions were prepared, with one containing 50 mM, and the plants were irrigated with these solutions until the end of the experiment. For the salinity treatment, stressed plants were irrigated twice a week with 1× Hoagland solution supplemented with 50 mM NaCl until the end of the experiment, while non-stressed plants were solely irrigated with 1× Hoagland solution without NaCl.

The experimental design was a split-split plot with three replicates. Each replicate included two main plots for salinity treatments (non-stressed and stressed), with each plot containing two subplots for genotypes (Tamaroi and Line 5004). Within each subplot area, four bacteria inoculation treatments (non-inoculated plants and those inoculated with *S. rhizophila*, *P. jordanii* strain G34, and *O. jordanicus* strain GSFE11) were randomly distributed.

### 4.4. Measurements and Data Analysis of Greenhouse Experiment

At the time of termination of the greenhouse experiment, the entire aboveground plant parts for each treatment were harvested and the following data were noted: total plant weight (TPW: g) was quantified (using Highland^®^ Precision Balance, Model: HCB 602aM, Adam Equipment Inc., Oxford, CT, USA) as the dry mass of the aboveground parts. Spike weight (SW: g) was determined by weighing the spikes derived from the harvested plants. Grain weight per plant (GWP: g) was assessed by threshing the spikes from each plant, cleaning the grains from the chaff, and subsequently weighing them. Spike number (SN) represented the count of spikes per pot. Spikelet number (SplN) denoted the average number of spikelets counted on three main spikes harvested from each pot. Grains per spike (GpS) were calculated by harvesting grains from three spikes, counting them, and determining the grains per spike by dividing the total grains by three. Thousand kernel weight (TKW: g) and kernel characteristics traits, including length (KL), width (KW), and area (KA), were evaluated by harvesting grains from three spikes and subjecting them to analysis using a MARVIN grain analyzer (GTA Sensorik GmbH, Bielefeld, Germany).

For the determination of nutrient levels (expressed in % dry weight (D.M.)) in treated plants, dried leaves (0.1 g) were ground into fine powder and analyzed following the method outlined by [44]. The finely ground sample of each treatment was placed in a 50 mL flask containing H_2_SO_4_ and the mixture was then boiled until the sample was fully digested. Thereafter, to each sample, an aliquot of 50 mL of distilled water was added, and the sample was then properly filtered. Thereafter, the filtrate was used to determine the concentrations of Ca^2+^ and Mg^2+^ by atomic absorption spectroscopy (Analyst 200, PerkinElmer, Waltham, MA, USA). For the determination of Na^+^ and K^+^, a flame photometer (Jenway, PFP7, London, UK) was used as described previously [45].

For statistical analysis of data for each variable, the GenStat statistical software (Release 16.1, 2013; VSN International Ltd., Hemel Hempstead, UK) was used. A combined ANOVA was worked out following the split-split plot design. All means within each variable were compared using the least significant difference test (LSD) at the 5% probability level. The corrplot package of the R software (version 2023.09.1+494) was employed to work out the Pearson correlation coefficient. 

### 4.5. Field Experiment

Field experiments were conducted across two agricultural research stations in Jordan during the growing seasons of 2019–2020 and 2020–2021. The first station, Jubeiha Agricultural Research Station at the University of Jordan (JU) campus, Amman, Jordan (32°00′40″ N, 35°52′24″ E; elevation: 990 m), represents a semi-humid area with a mean annual rainfall of 521 mm. The texture of the soil of this site is clay loam with a salt concentration of 0.58 dS.m^−1^, pH 7.87, and organic matter 2.41%. The second station, Al-Khalediyah (KD) Agriculture Salinity Research Station-National Agricultural Research (NARC) located at Al-Khalediyah, Mafraq, Jordan; 32°10′02.7″ N, 36°17′31.8″ E; elevation: 588 m), characterized by a mean annual rainfall of 130 mm, soil texture clay loam with salt level of 3.69 dS.m^−1^, pH 7.72, and organic matter 1.52%, represents an arid and saline area. In the JU field trial, the durum wheat genotypes were grown under natural rainfed conditions, receiving 742.90 mm of rainfall in 2020 and 381.00 mm in 2021. For the KD trial, where rainfall in 2020 was 141.40 mm and in 2021 was 102 mm, supplementary irrigation through the drip irrigation system was provided from a saline water resource (EC: 7.57 dS.m^−1^).

A split-plot design with three replicates was implemented. Each replicate comprised four main plots for bacterial inoculation treatments (*S. rhizophila*, *P. jordanii* strain G34, and *O. jordanicus* strain GSFE11), with each main plot containing two subplots for the two genotypes (Tamaroi and Line 5004 (carrying *Nax2*)); all treatments were randomly distributed. Within the subplot, the tested genotypes were randomly distributed, each sown in four adjacent rows (1 m length, 0.3 m apart), with a 0.5 m spacing between the two treated genotypes within each subplot. Seeding rates were adjusted based on a seed germination test (90% germination) to achieve a plant density of 50 plants/row (approximately 150 plants/m^2^). All field trials experienced a conventional tillage system. All standard agricultural practices such as fertilizer application, pesticide application for disease and weed control were followed. Weather stations recorded rainfall frequency and intensity as well as temperatures during the growing seasons. For soil testing, three samples were collected from each location and analyzed for physico-chemical properties, including soil texture, organic matter, electrical conductivity (EC), and pH, as previously described [29].

### 4.6. Measurements and Data Analysis of Field Experiment

At the end of each field trial, plants were harvested when reached the harvest maturity stage (no green tissue remained) and the following measurements were recorded: total weight (TW: g/m^2^) worked out as the whole dry mass of all harvested plants; for grain weight (GW: g/m^2^), all spikes were separated, threshed and cleaned and the clean grains were weighed; spike number (SN) from all harvested plants; straw weight (StW: g/m^2^) was derived by deducting GW from the TW; spike length (SL: cm): as the mean length of 10 spikes; spikelet number (SplN): as the average number of spikelets counted on 10 spikes; grains per spike (GpS) were calculated by dividing grain number on the total spike number; and harvest index (HI: %) was calculated by dividing GW over TW multiplied by 100. For 1000 kernel weight (TKW: g), kernel length (mm), width (mm), area (mm^2^), circularity (mm) and length/width ratio, over 50 grains for each treatment were harvested and analyzed using the MARVIN grain analyzer (GTA Sensorik GmbH, Germany).

For the determination of protein content (%), moisture content (%), fat content (%), fiber content (%), ash content (%), starch (%) and dry matter content (%) in the harvested grains, the NIRS™ DS2500 feed analyzer (Foss NIR Systems, Silver Spring, MD, USA) was used. 

## Data Availability

Data are contained within the article and Supplementary Materials.

## Supplementary Materials

The following supporting information can be downloaded at: https://www.mdpi.com/article/10.3390/plants13091179/s1, Table S1: Mean squares from the combined analysis of variance of tested traits from the greenhouse experiment; Table S2: Mean values of 10 tested agronomic traits, nutrient levels and K+:Na+ ratio from the greenhouse experiment; Table S3: Mean squares from the combined analysis of variance of tested traits from the field trials; Table S4: Mean values of 12 tested agronomic traits and six quality traits from the field experiment; Figure S1: Growth of two durum wheat genotypes (Tamaroi and Line 5004) inoculated with different types of PGPR in KD20 environment under saline conditions.
