# Supplementary figures and images for: Halotolerant Endophytic Bacteria Regulate Growth and Field Performance of Two Durum Wheat Genotypes with Contrasting Salinity Tolerance Potential
Source: Plants (Basel). 2024 Apr 23;13(9):1179. doi: 10.3390/plants13091179 (PMC11085664; doi:10.3390/plants13091179)

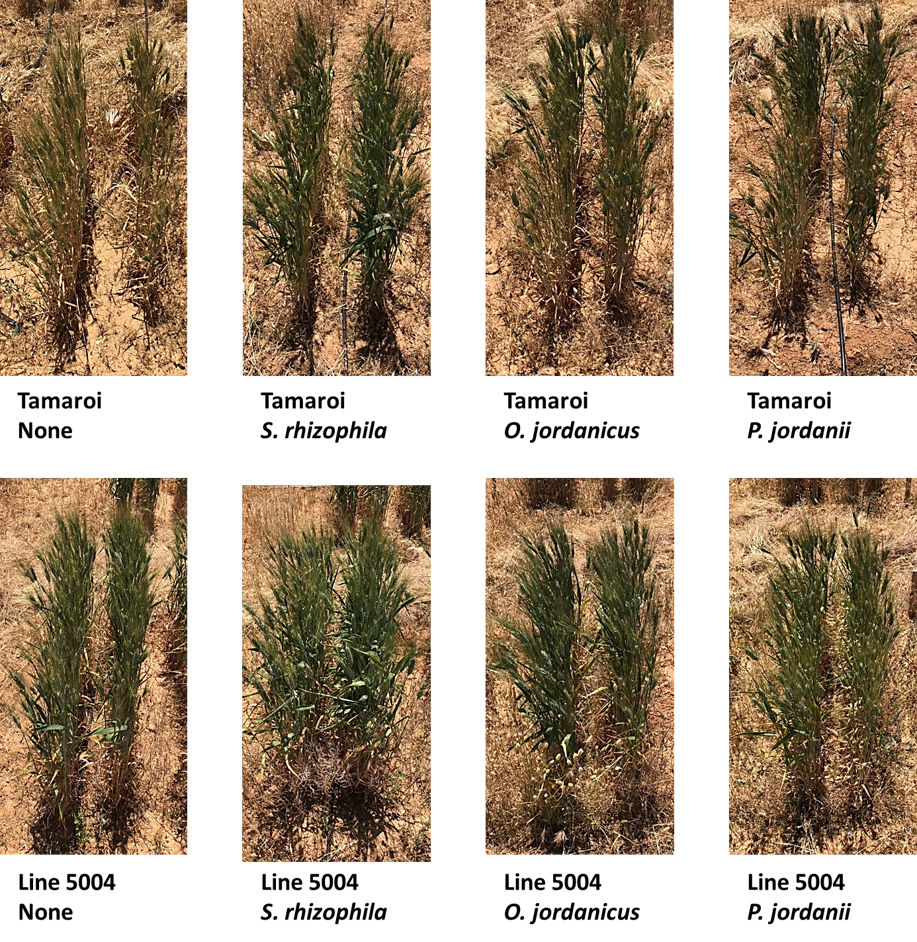

Supplement: Supplementary file 1 [file plants-13-01179-s001.zip › Figure S1.png]
